# Coordinating risk communication and community engagement for a better COVID-19 response in Eastern and Southern Africa

**DOI:** 10.11604/pamj.supp.2022.41.2.28150

**Published:** 2022-03-29

**Authors:** Charles Nelson Kakaire, Sharon Reader

**Affiliations:** 1 UNICEF Eastern and Southern Africa Regional Office, Nairobi, Kenya,; 2 International Federation of Red Cross and Red Crescent Societies Africa Region, Nairobi, Kenya

**Keywords:** Risk communication, community engagement, coordination, COVID-19

## Abstract

Risk Communication and Community Engagement (RCCE) is crucial for effective public health emergency response, with coordination of RCCE essential to avoiding duplication, resource wastage and possible confusion at community level. We describe the structure and operational modalities of the regional RCCE coordination mechanism for COVID-19 in Eastern and Southern Africa since the declaration of the first cases in countries in the region in March 2020. Under the co-leadership of UNICEF and the International Federation of Red Cross and Red Crescent Societies (IFRC), more than 30 agencies including UN agencies, Non-Government organisations, media and interfaith councils shared information on their interventions and support to the regional COVID-19 response. The technical working group has facilitated the development of joint guidance and reports. The group also shared monthly community feedback reports, Fact sheets, Theme specific Guidance Notes, media webinars and Social science evidence reviews from the sub-working groups. The Bill and Melinda Gates Foundation provided complementary resources to strengthen the regional coordination and tailored support to country RCCE response processes. This manuscript documents a regional approach to RCCE coordination for public health emergency response for potential replication and knowledge to inform and guide future RCCE for preparedness and response at regional level.

## Brief

### Introduction

WHO Regional Office for Africa´s emergency programme on March 2, 2020 in Nairobi, Kenya called a meeting of Health Partners which established a COVID-19 coordination mechanism for the East and Southern Africa region. The emphasis was on the importance of ramping up regional readiness for COVID-19 and partners´ commitment and support for a joint coordination mechanism for response planning and implementation [1]. The health partners established technical working groups in line with the standard response pillars, including Risk Communication and Community engagement. The RCCE Working group coordinated partners´ response, developed terms of reference, defined the membership and agreed on operational modalities. With a membership size of about 30 agencies including UN agencies, media organizations, interfaith council and non-government organization, a standing agenda was considered important. A regional coordination is essential for an effective response to disease outbreaks and especially so for a pandemic. We share the RCCE coordination approach adopted in Eastern and Southern Africa for potential replication and to inform future RCCE for preparedness and response.

### Information management using the READY Platform

Information about the activities of the group were published on the READY initiative (an inter-agency platform supporting NGO capacity to respond to major outbreaks of infectious disease with epidemic or pandemic potential) website [2]. The platform content received more than 3000 visits in the first two months and served the initial information needs of all members of the group. The platform also provided space for Partner mapping, showing which RCCE Partners, were implementing what activities, in which locations within the region.

### Collecting, analyzing and responding to community feedback

A subgroup was created to collate and analyze community feedback trends [3] from the member countries and agencies concerning citizen perspectives about COVID-19 using a standard google form. The subgroup meets fortnightly to discuss, analyze and categorize the concerns raised, develop recommendations, and determine the appropriate agencies and/or working groups to address them at either the regional or country level. The community feedback is shared with all partners and agencies and used for planning and refining intervention activities including community education, concerns related to case management, psychosocial support and infection prevention and control.

### Media engagement

One of the actions from community feedback group has been to engage the media as partner in the dissemination of correct information and dispelling of rumors and concerns from the communities. The taskforce meets twice monthly, to review the trends and recommendations from the community feedback report. The taskforce engages the media in a regular briefing to share community concerns, current scientific development and state of knowledge about the virus and the gaps in knowledge. Three media activities were held between March and November.

### Evidence-informed response

A social science research subgroup was created to review available data and community feedback that could be used to guide the response. In collaboration with the Social Science in Humanitarian Action Platform (SSHAP) [4], the group identifies gaps in knowledge and evidence needs and reviews secondary data for addressing relevant evidence gaps. The findings are published as research briefs.

### Linkages with other working groups

The RCCE working group collaborates with the surveillance technical working group and the case management technical working group which encompasses case management, mental health and psychosocial support, and Infection Prevention and Control. This ensures cross learning and information sharing, addressing issues related to risk communication and community engagement within the working groups.

### The collective service for RCCE

A global inter-agency mechanism to harmonize and strengthen the different regional RCCE working groups was established in June 2020 under the co-leadership of UNICEF, WHO and IFRC. Through dedicated human resource, the *Collective service* [5] supports the day to day operations of the RCCE working group, ensuring follow up of actions, accountability and collective working across the COVID-19 RCCE response at global and regional level.

### Conclusion

The RCCE partnership coordination at regional level was useful for instituting a collective approach to the COVID-19 response. The approach brings together the combined regional expertise for a region-wide response taking advantage of the synergy, complementarity and knowledge sharing that is inherent in collaboration. The platform encourages openness and trust which is necessary for responding to the pandemic.
